# Non-coding RNAs: emerging players in cardiomyocyte proliferation and cardiac regeneration

**DOI:** 10.1007/s00395-020-0816-0

**Published:** 2020-08-03

**Authors:** Naisam Abbas, Filippo Perbellini, Thomas Thum

**Affiliations:** Institute of Molecular and Translational Therapeutic Strategies (IMTTS), Hannover Medical School, Hanover, Germany

**Keywords:** Heart regeneration, Cardiomyocyte proliferation, MicroRNAs, lncRNAs, circRNAs

## Abstract

Soon after birth, the regenerative capacity of the mammalian heart is lost, cardiomyocytes withdraw from the cell cycle and demonstrate a minimal proliferation rate. Despite improved treatment and reperfusion strategies, the uncompensated cardiomyocyte loss during injury and disease results in cardiac remodeling and subsequent heart failure. The promising field of regenerative medicine aims to restore both the structure and function of damaged tissue through modulation of cellular processes and regulatory mechanisms involved in cardiac cell cycle arrest to boost cardiomyocyte proliferation. Non-coding RNAs (ncRNAs), such as microRNAs (miRNAs), long non-coding RNAs (lncRNAs), and circular RNAs (circRNAs) are functional RNA molecules with no protein-coding function that have been reported to engage in cardiac regeneration and repair. In this review, we summarize the current understanding of both the biological functions and molecular mechanisms of ncRNAs involved in cardiomyocyte proliferation. Furthermore, we discuss their impact on the structure and contractile function of the heart in health and disease and their application for therapeutic interventions.

## Introduction

Heart failure is a burgeoning health problem worldwide. Often a consequence of cardiovascular events, heart failure has become a major cause of morbidity and mortality and its incidence is expected to grow further in the future [137]. Upon cardiac injury, the adult mammalian heart does not regenerate sufficiently to compensate for the lost myocardium, resulting in a cardiomyocyte deficiency, and hence a lack of functional recovery of the infarcted or failing heart. Therefore, there is an unmet need to device effective regenerative strategies to induce cardiomyocyte renewal and replace the post-infarct fibrotic tissue with contractile muscle cells.

For many years, the human heart has been considered a post-mitotic and fully differentiated organ; this paradigm, however, has been challenged since various studies have reported a residual capacity of cardiomyocytes to proliferate after birth [8, 120]. This process has been shown to occur at a low rate and it progressively and continuously declines with age so much so that in a 60 years old human heart it does not exceed 0.5% [8]. On the contrary, other species such as zebrafish and newt can completely regenerate their hearts after myocardial injury by activating cardiomyocyte proliferation [4, 71]. Similarly, neonatal mammals such as rodents and pigs show some degree of regenerative capacity after injury which is lost during the first week after birth [54, 112, 166]. A human case report has recently suggested that a similar capability could be present in humans and demonstrated complete recovery of a 1-day-old newborn following a severe cardiac injury caused by myocardial infarction (MI) [55]. Taken together, these examples highlight the fact that cardiomyocytes harbor endogenous proliferative capabilities, suggesting that these mechanisms could be reactivated to boost proliferation in adult human cardiac tissue, thus providing new possibilities to be exploited for novel therapeutic strategies.

Non-coding RNA (ncRNA) account for the vast majority of the transcribed genome [28]. Unlike messenger RNA (mRNA), these sequences do not encode proteins, but rather act as regulators in the epigenetic, post-transcriptional, and translational coordination of gene expression [50]. In recent years, the reduced cost of next-generation sequencing technologies has led to an explosion of newly identified ncRNAs, and accumulating evidence has provided a more comprehensive knowledge of their biological functions [35]. Interestingly, a large number of ncRNAs seem to be cardiac specific [102], and different classes of ncRNAs have been linked to cardiac regeneration [108]. Gain- and loss-of-function approaches demonstrated an essential role of various ncRNAs in regulating the proliferation of cardiomyocytes in both in vivo and in vitro models. These modulations were sufficient to promote cardiac regenerative and reparative responses, which were observed at the functional, structural, and cellular levels [41, 132]. Among the different classes of ncRNA, microRNAs (miRNA) were the first to be identified as early as 1993 [79], thus they had a head start in regenerative research. So far, numerous miRNAs have been established for their role and mechanism in cardiac regeneration and their capability to promote cardiomyocyte proliferation^10^. On the contrary, long non-coding RNA (lncRNA) and circular RNA (circRNA) have only recently emerged as potential targets to boost cardiomyocyte proliferation. The aim of this review is to provide an overview of the current understanding of the roles that miRNAs, lncRNAs, and circRNAs play in the process of cardiomyocyte proliferation, as a means of cardiac regeneration. We discuss their potential to induce division of cardiomyocytes both in vitro and in vivo, and to attenuate tissue remodeling in terms of function and structure following cardiac injury. Finally, current limitations and future directions for successful translational research are discussed.

## Cardiomyocyte proliferation and heart regeneration

The mammalian heart responds to myocardial infarction and consecutive cell death by the progressive replacement of the injured myocardium with a meshwork of extracellular matrix and proliferating cells that forms a scar. This wound healing process involves an inflammatory response [48] and the simultaneous activation of local stromal cells [13]. The fibrotic scar has been shown to be essential to safeguard the cardiac wall and prevent the fatal rupture of the dead myocardium [45]. Yet, the progressive accumulation of extracellular matrix and associated tissue remodeling have profound effects on the cardiac function and structure [13]. The remaining cardiomyocytes experience abnormal mechanical stress and develop compensatory hypertrophy [77] and reorganization of their sarcomere structure [38]. Additionally, cardiomyocyte remodeling results in impaired intracellular calcium handling and altered contraction and relaxation [60]. These major alterations in cardiac function and structure have led to the emergence of novel regenerative strategies that aim to attenuate the progression of myocardial remodeling and repopulate the fibrotic scar with cardiomyocytes which are mechanically and electrically integrated with the surrounding tissue. Embryonic stem cells (ESC) and induced pluripotent stem cells (iPSC) have been extensively studied for cell therapy as an exogenous origin of new cardiomyocytes. Either delivered by direct injection or cardiac patch, pluripotent stem cell-derived cardiomyocytes were shown to engraft in infarcted hearts and promote beneficial effects on regional and global cardiac function [21, 25, 93, 123]. Nevertheless, challenges like the immature phenotype and poor survival of cardiomyocytes [99], risk for tumorgenicity [100], and increased propensity to develop arrhythmic events [158] need to be overcome before their translation to clinical use. An alternative is direct reprogramming which aims to convert resident cardiac fibroblasts into cardiomyocytes in situ. Several small molecules, transcription factors, and ncRNAs have been identified and used to mediate the direct trans-differentiation [32, 65]. This evidence, however, is still not sufficiently robust to determine whether this approach can generate the large number of cardiomyocytes needed to replenish the lost myocardium. Finally, cardiomyocyte cell cycle re-activation and stimulation of their proliferative capacity seem to be the most direct approach. Accumulating data are offering various strategies to trigger this process and generate new cells. Despite some challenges which will be discussed later in the text, this is an exciting avenue with great translational potential.

### Cardiomyocyte proliferation in the steady-state

During the embryonic development, cardiomyocytes are generated by two main mechanisms: (1) the commitment and differentiation of cardiogenic precursor cells derived from the mesoderm cell layer, and (2) the proliferation of existing cardiomyocytes. The former is dominant throughout the formation and expansion of the early heart tubes [14], while the development of the heart tubes into the four-chambered heart shape is driven mainly by the proliferation of differentiated myocytes [126]. Later phases of the heart development, such as trabeculation and wall maturation, are also dependent predominantly on cardiomyocyte division [51, 52]. After birth, cardiac growth in mammals shifts abruptly from hyperplasia to hypertrophy, and eventually, an almost complete mitotic arrest occurs around postnatal day 4 (P4) in mice [82]. In this brief window of time, mammalian newborns retain a transient capacity for cardiomyocyte renewal. Surgical resection of the cardiac apex in P1 mice was shown to trigger a robust upregulation of myocyte proliferation and near-complete regeneration of the injury site by day 21 [112]. The cell cycle withdrawal after birth was shown to be mediated by a complex of regulatory factors which can be extrinsic, e.g., oxygen level, mechanical forces, and macrophage-associated factors, or intrinsic, e.g., Hippo/Yap pathway, Neuregulin/ErbB2 pathway, Meis1, E2F and miR-15 [113, 140]. The potential contribution of these factors to cardiac regeneration, however, is still not fully determined and currently they are the subject of intensive research.

Non-mammalian species, like teleost fish and amphibians, have a pronounced regenerative capacity which has also been exploited for translational research. Studies on zebrafish have identified many factors such as JAK/STAT pathway, retinoic acid, Notch, and non-coding RNAs such as miR-133, miR-99/100 and Let-7a/c which are involved in cytokinesis [1, 36, 74, 155, 164]. The modulation of miR-99/100 and Let-7a/c and their target genes SMARCA5 and FTNB was shown to induce cardiomyocyte dedifferentiation and proliferation in mammals suggesting that the molecular machinery responsible for zebrafish heart regeneration can be effectively utilized on mammalian hearts [1]. Although these data are promising, other molecular mechanisms have failed to achieve the same goal [114], indicating the need for further investigations to better understand the applicability and limitations of this approach.

### Current approaches to induce cardiomyocyte proliferation and pathways involved

Several physiological conditions, such as oxygen level and mechanical load, have been reported to push cardiomyocytes into mitosis. By measuring ^15^N-thymidine labeling of DNA synthesis, Vujic et al. [139] have reported increased cardiomyocyte proliferation in both healthy and MI adult mice which had to perform exercise for 8 weeks [139], suggesting that exercise promotes cell cycle re-entry [139]. In addition to exercise, oxygen levels are also known to play an important role in cell cycle withdrawal. One of the major changes that newborns experience after birth is the abrupt increase in oxygen levels. An increase in reactive oxygen species (ROS) production and consequent activation of DNA damage responses have a pivotal role in cardiomyocyte cell cycle arrest [115]. On the contrary, exposure to severe systemic hypoxemia of 7% O_2_ for 2 weeks in adult mice was shown to reduce ROS generation and reactivate the cell cycle in cardiomyocytes. Additionally, when hypoxia was applied to post-MI mice, it was able to induce cardiomyocyte proliferation accompanied by improved cardiac function, vascular expansion, and decreased fibrosis [97]. Mechanical unloading of the left ventricle has also been reported to induce cardiomyocyte proliferation. Increased mechanical stress induced in volume overload models activates mitochondrial biogenesis, which increases ROS production; thus, similar to hyperoxemia it results in the activation of DNA damage responses [109]. Canseco et al. [19] examined the effects of human ventricular unloading after implantation of left ventricular assist devices (LVADs) by comparing left ventricular samples collected at the time of LVAD implantation (pre-LVAD) and at the time of explantation (post-LVAD). Their findings demonstrated that post-LVAD hearts had increased cardiomyocyte proliferation, suggesting that mechanical unloading promotes the cell cycle progression.

The modulation of several coordinated signal transduction pathways is known to reactivate the cell cycle in adults [37]. The Hippo/YAP transduction pathway is an evolutionarily conserved regulator of organ size in different tissues [159]. In the heart, it was shown to be a key regulator of cardiomyocyte proliferation during heart development and cardiac regeneration in the early postnatal stage [138, 152]. Following the activation of the cascade, the transcriptional co-activators Yes-associated protein (YAP) and transcriptional coactivator with PDZ-binding motif (TAZ) become phosphorylated and inactive, which results in their dissociation from transcription factors that normally drive cell division [161]. Blocking the Hippo/YAP pathway by targeting its components has been shown as an effective approach to boost cardiomyocyte proliferation accompanied by improvement in total cardiac function [56, 78, 152]. Meis1 is another transcription factor that was linked to cardiac myocyte proliferation during development and in the early postnatal regenerative phases [87, 127]. Meis1 facilitates the cell cycle withdrawal by activating the expression of the cyclin-dependent kinase (CDK) inhibitors p15, p16, and p21 [87]. The transcription factor TBx20 has also direct interaction with Meis1 [150]. The downregulation of Meis1 was shown to reactivate cardiomyocyte mitosis in the adult heart with no deleterious effect on cardiac function [87]; however, whether this ameliorates the cardiac structure and function in the post-MI setting still needs to be determined. Several paracrine factors have been reported to regulate cardiomyocyte proliferation. Neuregulin-1 (NRG-1) is secreted from microvascular endothelial cells and acts on cardiomyocytes through its receptors ErbB2 and ErbB4 [40]. Activation of NRG-ErbB pathway either by overexpression of ErbB4 or by using a recombinant NRG-1 was reported to improve cardiac function and decrease post-MI fibrosis [10]. Moreover, clinical trials reported a favorable effect of recombinant NRG-1 on the cardiac function in heart failure patients [44, 67]. These findings however were not confirmed in a mouse study performed by Reuter et al. [117]. Experimental evidence indicated that NRG-1 did not activate cardiomyocyte DNA synthesis in either healthy or infarcted mice, thus suggesting that improved cardiac structure and function likely occurred independently of apparent myocardial regeneration. More work will be required to better understand NRG-1 cardiac reparative effects and its role in cardiomyocyte proliferation.

Finally, ncRNAs are also emerging as common targets to modulate cardiomyocyte proliferation. These molecules form a complex regulatory network with other molecular elements such as mRNAs and proteins which regulate cardiomyocyte cycle progression. Here we discuss miRNAs, lncRNAs, and circRNAs with such proliferative capacity and present their underlying mechanism.

## MicroRNAs as targets for cardiomyocyte proliferation

**Fig. 1:**
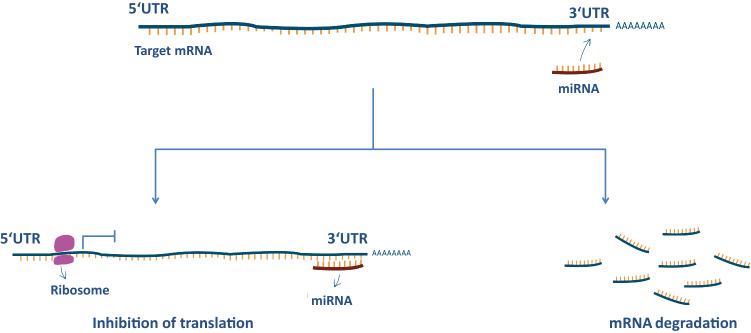
miRNA mode of action. In most cases, miRNAs bind to a specific sequence at the 3′ UTR of their target mRNAs. This binding results in either translational repression or degradation of the respective mRNA target

MicroRNAs are small (≈22 peptides), single-stranded, noncoding RNAs that regulate gene expression at the post-transcription stage. These molecules repress protein expression by interacting with completely or partially complementary sequences of the 3′-untranslated regions (3′-UTR) of mRNAs. This binding leads to degradation or translational repression of the targeted mRNA [6] (Fig. 1). A single miRNA can target several to hundreds of genes, whereas various miRNAs can collectively target one mRNA [124]. These combinatorial interactions allow for a complex fine-tuning of several regulatory processes. It is not surprising that disruption of miRNA biogenesis and function contributes to many human diseases, including cancer, cardiovascular diseases, and neurological disorders [125, 130, 163].

Since the initial discovery of miRNAs in 1993 in the nematode *Caenorhabditis elegans* [80], thousands of miRNA genes have been identified in various species, among these more than 1500 were identified in humans [73]. It is estimated that two-thirds of the human protein-coding genes have miRNA target sites in their 3′ UTR; thus, they are potentially regulated by these molecules in both health and disease [39]. Numerous miRNAs have been identified in the cardiovascular system and were shown to control a wide range of biological processes, including cardiac repair, lineage commitment, proliferation, and cardiomyocyte survival [131]. miRNAs have been studied in the field of cardiac regeneration and have been found to tightly control cell cycle re-entry in cardiomyocytes. Here, we summarize a group of recently discovered miRNAs in the field and we outline their mechanism of action and potential gene targets (Table 1).

**Table 1:** A summary of the in vitro and in vivo effects of miRNAs on cardiomyocyte proliferation and their mechanism of action

miRNA	In vitro effects	In vivo effects	Mechanism of action
*Positive regulators*			
miR-199a	CM proliferation ↑ CM number ↑	CM proliferation ↑ CM area ↔ post-MI cardiac function ↑ post-MI scar area ↓ (prolonged overexpression) sudden death ↑	Downregulates various proliferation-associated genes; e.g., Meis2, Rb1 and P16, HOMER1, HOPX, CIRC5 Targets regulators of the Hippo pathway (TAOK1 and β-TrCP) Downregulates cofilin 2
miR-590a	CM proliferation ↑ CM number ↑	CM proliferation ↑ CM area ↔ post-MI cardiac function ↑ post-MI scar area ↓	Downregulates genes HOMER1, HOPX, CIRC5 Activates YAP
miR-1825	CM proliferation ↑ CM number ↑ oxidative stress ↓ DNA damage ↓	CM proliferation ↑ CM area ↓ post-MI cardiac function ↑ post-MI scar area ↓	Downregulates mitochondrial NDUFA10 Increases miR-199a expression Downregulates cofilin 2
miR-31a	CM proliferation ↑ CM number ↑ PCNA expression ↑	CM proliferation ↑ PCNA expression ↑	Downregulates RhoBTB1
miR-302–367	CM proliferation ↑	CM proliferation ↑ CM number ↑ Cardiomegaly CM area ↓ expression of genes associated with proliferation and negative regulation of cell differentiation ↑ post-MI scar area ↓ (prolonged overexpression) post-MI cardiac function ↓ (transient overexpression) post-MI cardiac function ↑	Targets genes of the Hippo transduction pathway (MST1, LATS2, MOB1B) Downregulates cofilin 2
miR-294	CM proliferation ↑ metabolic demand ↑	Post-MI overall survival ↑ post-MI cardiac function ↑ post-MI scar area ↓ post-MI apoptosis ↓ post-MI CM size ↔ post-MI expression of hypertrophy markers ↓	Targets Wee1 Increases CDK1-cyclin B1 complex activity
miR-499	CM proliferation ↑ CM viability ↑ CM apoptosis ↓		Downregulates cyclin D1 Targets SOX6
miR-17–92		CM proliferation ↑ CM number ↑ post-MI CM number ↑ post-MI cardiac function ↑	Represses the expression of PTEN
*Negative regulators*			
miR-1	CM proliferation ↓ CM number ↓ CM viability ↓		Downregulates cyclin D1
miR-34a	CM proliferation ↓	Post-MI CM proliferation ↓ post-MI CM apoptosis ↑ post-MI fibrosis ↑	Targets BCL2, cyclin D1 and SIRT1
miR-29a	CM proliferation ↓		Targets cyclin D2
miR-133a		Cardiac fibrosis ↓ CM proliferation ↓ CM apoptosis ↓ LV wall thickness ↓	Downregulates SRF and cyclin D2 Represses SRF and smooth muscle gene expression
miR-let 7i 5p	CM proliferation ↓ CM number ↓	CM proliferation ↓ post-MI cardiac function ↓ post-MI fibrosis ↑ post-MI apoptosis ↑	Targets E2F2 and cyclin D2
miR-128		Cardiomegaly CM proliferation ↓ CM cross-sectional area ↑ cardiac function ↓ post-MI fibrosis ↑ post-MI cardiac function ↓ heart regeneration in neonates ↓	Targets SUZ12 Inhibition of miR-128 upregulates positive cell cycle regulators, e.g., cyclin E and CDK2
miR-29b	CM proliferation ↓	CM proliferation ↓ (zebrafish)	Targets NOTCH2

*miR* microRNA;* CM* cardiomyocyte;* MI* myocardial infarction;* PCNA* Proliferating cell nuclear antigen

**Fig. 2:**
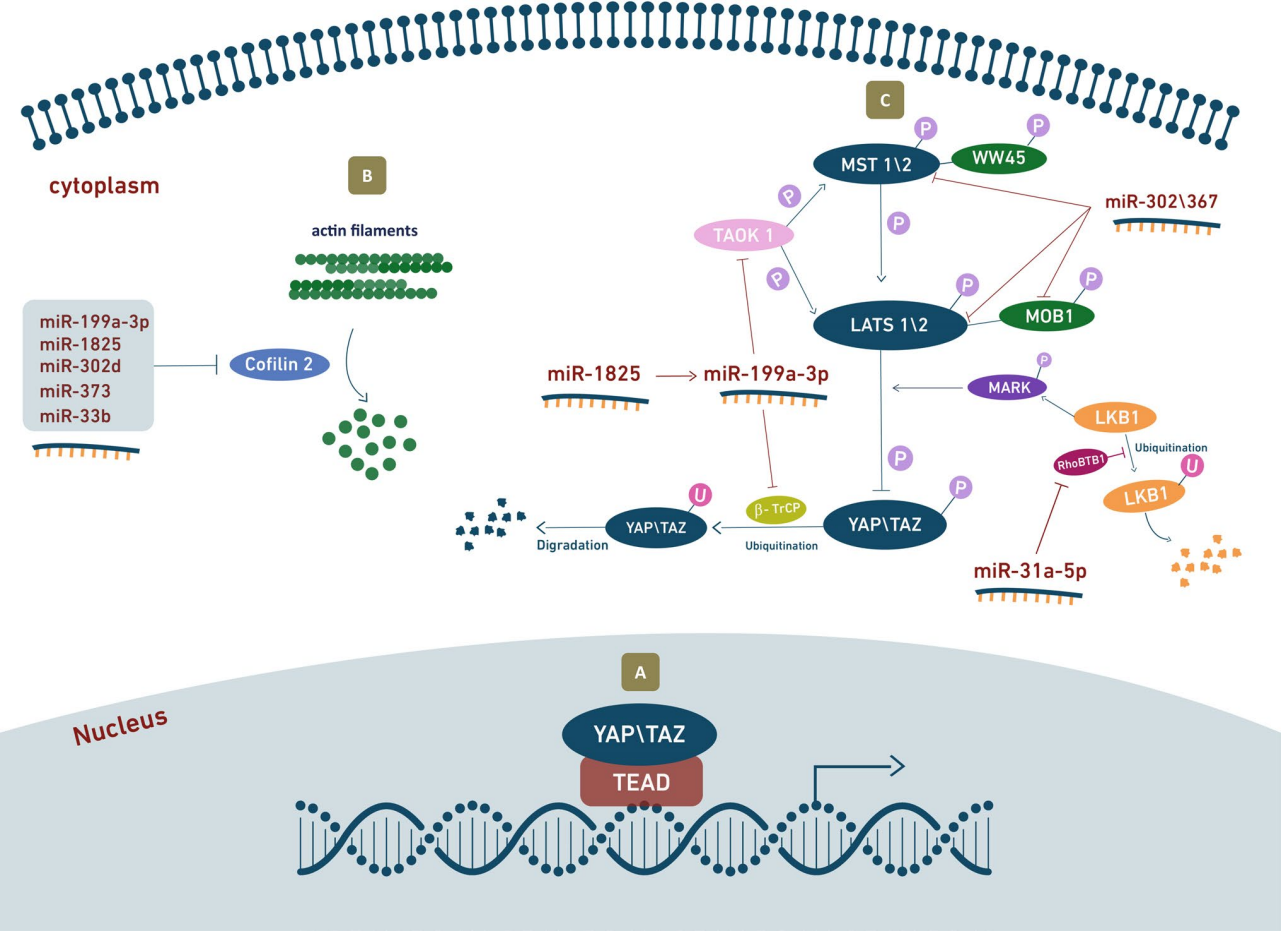
Hippo pathway mediates the activity of miRNAs inducing cardiomyocyte proliferation. **a** The active dephosphorylated form if YAP/TAZ localizes to the nucleus and associates with TEAD transcription factors to drive cell proliferation genes expression. **b** miR-199a-3p, miR-1825, miR-302d, miR-373 and miR-33b downregulate cofilin 2, which disassembles actin filaments. The resulting cytoskeletal rearrangement leads to YAP activation and nuclear localization. **c** When Hippo signaling is on, MST1/2 activate LATS1/2 kinases, which in turn phosphorylate and inactivate the downstream effectors YAP and TAZ. miR-302/367 complex directly targets the expression of MST1, LATS2 and MOB1B, thereby blocking the Hippo signaling. miR-1825 activates miR-199a-3p, resulting in the downregulation of its target genes TAOK1 and β-TrCP, eventually leading to Hippo pathway repression and prevention of YAP degradation. miR-31a-5p downregulates RhoBTB1 and results in Hippo deactivation

In 2012, Eulalio et al. identified a large series of human miRNAs reported to induce cardiomyocyte proliferation in vitro [34]. The same group has recently shown that the ten most effective miRNAs converge in the regulation of the Hippo pathway [134]. This pathway is a highly conserved signal transduction cascade that was first identified in Drosophila [53, 129]. It comprises a wide network of components that integrate diverse signals to eventually regulate cell proliferation and control organ size [161]. Activation of the Hippo pathway results in the phosphorylation of the master transcriptional cofactor YAP, thus blocking its activity. On the contrary, when YAP is dephosphorylated, it localizes to the nucleus and associates with the transcriptional enhanced associate domain (TEAD) 1–4 transcription factors to drive gene expression and stimulate cell proliferation [162] (Fig. 2a). Consistently, YAP is an essential factor in early heart development [138] and it is currently one of the most important targets for cardiac regeneration [152]. The miRNAs that were investigated include human miR-590-3p, miR-199a-3p, members of the miR-302 family (miR-302d, miR-302c, and miR-373), miR-1825, miR-1248, miR-18a, miR-33b, and miR-30e, all of which were shown to significantly increase the dephosphorylated YAP levels in the nucleus and improve TEAD activity in vitro. These effects were prevented by the knockdown of YAP, suggesting it is an essential requisite to mediate the pro-proliferative outcome of the investigated miRNAs [134]. These findings were confirmed by a study performed on hiPSC-derived cardiomyocytes, which found that 84 out of 96 miRNAs that promote proliferation upon overexpression induced nuclear translocation of YAP, and most of these miRNAs (67/84) required YAP for their proliferative activity [27]. These miRNAs act through different pathways to induce YAP activation. Some were found to directly target components of the Hippo pathway, such as the kinases MST1/2 and LATS1/2, while others regulate YAP via other mechanisms. For example, an intriguing interplay between YAP activation and the cytoskeletal arrangement was reported [134]. In particular, miR-199a-3p, miR-1825, miR-302d, miR-373, and miR-33b were found to downregulate the protein cofilin 2 and, except for miR-33b, this was achieved by directly binding to the 3′ UTR of cofilin 2 mRNA. Cofilin 2 is an actin-regulatory protein that binds actin monomers and filaments, causing their depolymerization and preventing their re-assembly [47], thus suggesting that proliferation was induced by the modulation of the actin cytoskeleton network (Fig. 2b).

Among the investigated miRNAs, miR-199a-3p is one of the most potent promoters of cardiomyocyte proliferation and one of the first miRNAs shown to activate, rather than suppress the cardiac cell cycle [34]. Cardiomyocyte treatment with miR-199a-3p demonstrates downregulation of both the TAO kinase1 (TAOK1) and the E3 ubiquitin-ligase β-transducing repeat-containing protein (β-TrCP) [134] (Fig. 2c). The former phosphorylates and activates the MST and LATS kinases [11, 111], whereas the latter catalyzes YAP ubiquitination, resulting in its degradation [160]. Additionally, miR-199a-3p, along with miR-590a-3p, are known to target the genes HOMER1, which encodes a protein that modulates calcium signaling in the heart, and HOP homeobox (HOPX), which encodes a homeodomain protein that inhibits cardiomyocyte proliferation [34]. Both miR-199a-3p and miR-590a-3p are undetectable in the postnatal state, but when administered to isolated adult cardiomyocytes they are effective inducer of cell cycle re-entry [34]. These miRNAs were also tested using different delivery methods in vivo and have shown promising effects in both small and large mammals. Intracardiac delivery and vector-based overexpression of miR-199a-3p and miR-590a-3p in neonatal rodents resulted in ventricular wall thickening and increased cardiac myocyte proliferation with no signs of cardiomyocyte hypertrophy. In a post-MI adult mouse model, they also increased cardiomyocyte proliferation markers, decreased the infarct size and improved cardiac function [34]. Even a single intracardiac injection in mice, immediately after MI, led to persistent recovery of cardiac function [80]. Consistent with these experiments on small mammals, a study performed on pigs showed that expression of human miR-199a in infarcted porcine hearts can boost cardiac repair. This was determined by the marked increase in cardiomyocyte proliferation markers, improvements in both global and regional contractility, increased muscle mass, and reduced scar size. Of note, persistent and uncontrolled expression of this miRNA resulted in sudden arrhythmic death of most of the treated pigs, suggesting that the dosage of this therapy needs to be tightly controlled [41]. Taken together, these data suggest that both miR-199a-3p and miR-590a-3p are powerful regulators and valid candidates for future clinical trials.

miR-1825 is also one of the miRNAs that target cofilin 2 and induce YAP activation [134]. In addition, a recent study has demonstrated a second mechanism that involves miR-199a. The authors showed that miR-199a has significantly increased in adult cardiomyocytes post-miR-1825 transfection, indicating that miR-199a acts downstream of miR-1825 [104]. While it is well established that miRNAs act by binding to the 3′ UTR of mRNAs, there have been very few reports on miRNA targeting other miRNAs [18, 88, 89]. Through miR-199a, miR-1825 treatment downregulated the cell cycle inhibitory genes Meis2, Rb1, and P16 [104]. Consequently, the overexpression of miR-1825 in adult rat cardiomyocytes increased both their number and their proliferation markers. Furthermore, a reduction in mitochondrial number and a decrease in ROS and DNA damage were observed. In vivo, miR-1825 administration to P1 neonatal mice resulted in enlarged hearts and increased cardiomyocyte numbers. Prolonged delivery of miR-1825 following MI increased cardiomyocyte proliferation improved heart function and decreased scar size in adult mice [104].

Another important regulator of the Hippo pathway is the miRNA cluster miR302-367. This cluster is made of five miRNAs that are expressed in the heart during the early embryonic stage. Tian et al. [132] have shown that when this cluster is suppressed in mouse embryos, proliferation markers and expression of the cell cycle proliferation gene Ccnd1 were downregulated, as well as other genes involved in cell differentiation such as Nkx2.5, Gata4, Myh6, and Myh7. When overexpressed, miR302-367 increased cardiomyocyte proliferation and induced profound cardiac enlargement and cardiomegaly. Interestingly, although prolonged overexpression of miR302-367 in post-MI mice reduced fibrosis and improved cardiomyocyte proliferation, the treatment resulted in an overall altered cardiac function. On the contrary, a transient administration (over 1 week) resulted in a significant improvement in cardiac function. Experimental evidence suggested that miR302-367 can promote a proliferative state, but it also induced cardiomyocyte dedifferentiation. This dedifferentiation, however, can be avoided by short-term treatments. In addition to the actin-regulatory function of miR-302d, which is mediated by the interaction with cofilin 2 [134], miR302-367 inhibits the kinases Mst1, Lats2, and Mob1b. These are core components of the Hippo signaling cascade, whose inactivation eventually results in the active dephosphorylated form of YAP and, as a result, in a proliferative state [132].

Normally, miRNAs that function as positive regulators of cardiomyocyte proliferation are upregulated in the embryonic and fetal stages and downregulated afterward. On the contrary, miR-31a-5p was found to be significantly upregulated in P10 cardiomyocytes compared to P0, yet it was shown to activate the cardiomyocyte cell cycle [151]. This unusual pattern of differential expression was considered as a compensatory response. miR-31a-5p overexpression in neonatal rat ventricular myocytes (NRVM) and in rat neonates was sufficient to induce cardiomyocyte proliferation, assessed by immunostaining and the expression levels of proliferating cell nuclear antigen (PCNA) [151]. Bioinformatics analysis identified RhoBTB1 as the target gene of miR-31a-5p. RhoBTB1 is a tumor suppressor gene involved in different types of cancer [7, 90], but its role in cardiac pathologies is still unclear. Interestingly, a recent study also suggested a regulatory role of RhoBTB on the Hippo signaling [98]. This role was reported in Drosophila and human (HEK293T) cells and was shown to be mediated by ubiquitination-dependent regulation of LKB1 kinase [151]. LKB1 controls the Hippo pathway through phosphorylation of MARK kinases and thereby modulation of YAP activity [96].

Despite the prominence of the Hippo transduction pathway in miRNA-mediated cardiomyocyte proliferation, other pathways are also involved. The cyclin family is a key factor in the decision as to whether somatic cells become quiescent or resume active proliferation [42]. This mechanism is also highly regulated by miRNAs. For example, miRNAs miR-1 [43], miR-499 [84] and miR-34a [154] control cardiac myocyte proliferation through the regulation of cyclin D1, while miR-29a [20], miR-133a [86] and miR let-7i-5p [61] target cyclin D2. Moreover, miR-294 [12] and miR-128 [64] regulate cyclin B1 and cyclin E, respectively. Except for miR-499 and miR-29a, all these miRNAs have been reported to stimulate cardiomyocyte proliferation in vivo, indicating a high potential for successful translational applications. In addition to the cyclin proteins, phosphatase and tensin homolog (PTEN) is a tumor suppressor gene that can be the target for miRNAs promoting cardiac cell cycle progression. The miR-17–92 cluster, which directly targets PTEN, is known to be required and sufficient to induce cardiomyocyte proliferation. The overexpression of this cluster in adult animal hearts attenuated myocardial infarction-induced injury [23]. miR-29b-3p [153] and miR-34a [9] have been described to target the Notch signaling pathway which also plays an important role in the differentiation and proliferation of cardiomyocytes [107]. Of note, Notch is known to act downstream of Hippo, as suppression of Hippo is a potent activator of Notch signaling [66]. miR-29b-3p inactivates the NOTCH2 gene and its inhibition promoted cardiomyocyte proliferation in vitro and in vivo [153]. miR-34a, however, targets NOTCH1 and its regulator enzyme Pofut1 [9]. Inhibition of miR-34a reduced the scar size and improved cardiac function post-MI [154].

The data presented here propose a great potential of miRNAs as therapeutic targets in regenerative medicine. It is not surprising that miRNAs are gaining a lot of interest among pharmaceutical companies, which are dedicating significant efforts to develop efficient, safe, and easy-to-deliver miRNA products. Nevertheless, several obstacles remain to be overcome, the most important of which is the off-target biological effects which are complicated by the miRNA pleiotropic nature. Another important aspect is the fundamental genetic difference between humans and the animal models used for testing which further complicates the translatability of results, ultimately delaying the human clinical application. Furthermore, some miRNAs were shown to effectively stimulate the proliferation of adult cardiomyocytes by controlling the expression of target molecules, while the modulation of the respective targets was not sufficient to produce the same outcome. The overexpression of cyclin D1 in mice, for example, resulted in DNA replication and multinucleation of cardiomyocytes, but these cells did not proceed to cytokinesis and complete cell division [125]. These results suggest that miRNAs that target cyclin D1 also target other genes to successfully complete cell division. Moreover, activation of Notch signaling in zebrafish hearts unexpectedly suppressed cardiomyocyte proliferation and heart regeneration. This could be attributed to the over-stimulation of Notch signaling in the endocardium and/or epicardium which might lead to the production of paracrine signal that controls myocardial cell division [164]. These, however are speculations and a deeper understanding of miRNAs molecular targets and mechanisms are required to develop more efficient, effective, and safe therapeutic strategies.

## Long non-coding RNAs as targets for cardiomyocyte proliferation

lncRNAs are defined as RNA transcripts of > 200 nucleotides with no evidence for a protein-coding function [94]. Initially termed ‘mRNA-like noncoding RNA’ after its first discovery in 1999 [33], this subset of ncRNA has quickly gained popularity among researchers, and according to NONCODE database, 548,640 lncRNAs have already been annotated from 17 species [35]. From the human genome alone, 96,308 lncRNA transcripts are known, by far exceeding the number of protein-coding genes by about fivefold [118]. Unlike protein-coding genes and miRNAs, the majority of lncRNAs have poor interspecies sequence conservation [105], although it is speculated that conservation of their secondary structure might exist [69].

**Fig. 3:**
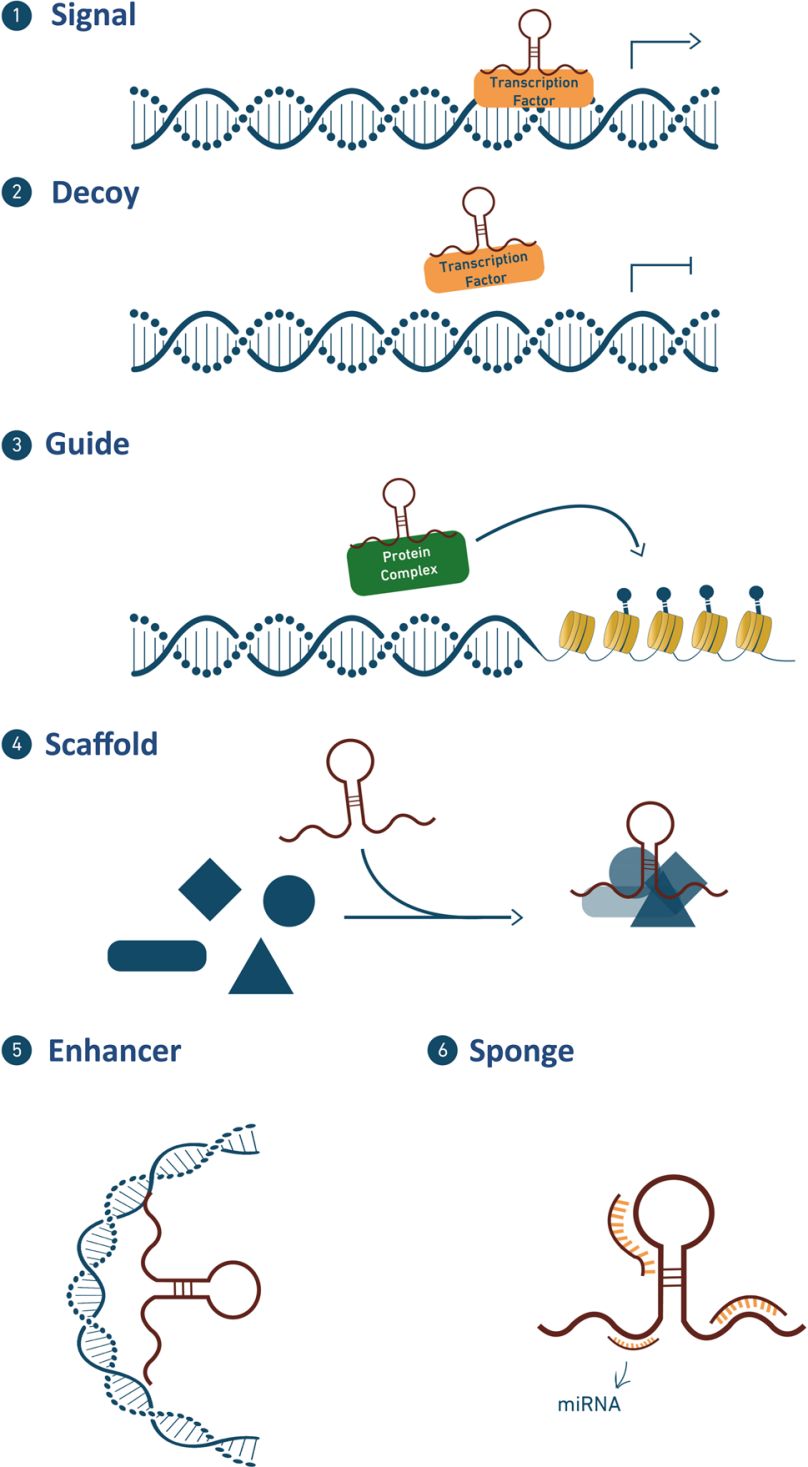
lncRNA modes of action. (1) signal—acts in response to stimuli, (2) decoy—sequesters transcription factors/protein complex, (3) guide—guides transcription factors/protein complex to a specific target site, (4) scaffold—brings together multi-protein complexes, (5) enhancer—induces chromosomal looping to increase association between enhancer and promoter regions, and (6) sponge—acts as a competing endogenous RNA (ceRNA) and sponge miRNAs

Based on genomic location and orientation to protein-coding genes, there are six major classes of lncRNAs: (1) sense—spanning multiple introns or exons within a gene, (2) sense intronic—located within one intron, (3) antisense—transcribed from the opposite strand of a gene, (4) bidirectional—located on the antisense strand within 1 kb of a promoter on the sense strand, (5) enhancer—located in an enhancer region and (6) intergenic—located between two genes [26]. Another type of classification relies on the cellular function of lncRNAs which are divided to signal, decoy, guide, scaffold, enhancer, or sponge lncRNA (Fig. 3) [5].

Accumulating evidence shows that lncRNAs play key roles in a large number of key biological processes, such as the circuitry controlling pluripotency and differentiation, immune responses, disease etiology, and chromosome dynamics [35]. In the cardiovascular system, lncRNAs are expressed in an abundance and it has been suggested that they compose a complex regulatory network governing the physiology (and pathology) of the heart. They are indeed involved in the regulation of various biological functions: for example, CARMEN [101], H19 [116] and Braveheart [76] play crucial roles in cardiomyocyte differentiation, Meg3 [149] and CARL [144] are essentially involved in apoptosis, and Chast [136] and Chaer [147] are associated with cardiac hypertrophy. Here, we highlight and summarize some of the recent developments and experimental findings related to the roles that different lncRNAs play in controlling cardiomyocyte proliferation and their implication in cardiac regenerative medicine (Table 2).

**Table 2:** A summary of the in vitro and in vivo effects of lncRNAs on cardiomyocyte prolfieration and their mechanism of action

lncRNA	In vitro effects	In vivo effects	Mechanism of action
*Positive regulators*			
NR_045363	CM proliferation ↑	CM proliferation ↑ Post-MI infarct area ↓ Post-MI cardiac function ↑	Sponge Sponges miR-216a Blocks JAK2/STAT3 pathway
Sirt1 AS lncRNA	CM proliferation ↑ CM apoptosis ↓ CM number ↑ CM cross-sectional area ↓	CM proliferation ↑ CM apoptosis ↓ CM cross-sectional area ↓ Post-MI overall survival ↑ Post-MI infarct size ↓ Post-MI cardiac function ↑	mRNA stabilization Stabilizes Sirt1 mRNA Increases Sirt1 protein levels
ECRAR	CM proliferation ↑ CM number ↑ CM volume ↔	CM proliferation ↑ Capillary and arteriole density ↑ Post-MI infarct size ↓ Post-MI fibrosis ↓ Post-MI cardiac function ↑	Signal Regulated by E2F1 Mediates ERK1/2 phosphorylation ERK1/2 increase the transcription of cell Cycle progression genes
*Negative regulators*			
CPR	CM proliferation ↓ CM number ↓	CM proliferation ↓ CM number ↓ Post-MI cardiac function ↓ Post-MI scar area ↓ Post-MI CM apoptosis ↑ Heart regeneration in neonates ↓	Guide Interacts directly with DNMT3A DNMT3A methylate MCM3 promoter MCM3 is downregulated
CRRL	CM proliferation ↓ CM cross-sectional area ↔	CM proliferation ↓ Post-MI cardiac function ↓ Post-MI scar area ↑ Post-MI fibrosis ↑	Sponge Sponges miR-199a-3p Activates HOPX Mediates Gata4 deacetylation
CAREL	CM proliferation ↓ (hiPSC-CM)	CM proliferation ↓ CM cross-sectional area ↑ Post-MI cardiac function ↑ Post-MI scar area ↓ Heart regeneration in neonates ↓	Sponge Sponges miR-296 Increases the expression of genes Trp53inp1 and Itm2a
DACH1	CM proliferation ↓ (hiPSC-CM)	CM proliferation↓ CM cross-sectional area ↑ Post-MI cardiac function ↑ Post-MI scar size ↓ Heart regeneration in neonates ↓	Decoy Inactivates PP1A Mediates YAP1 phosphorylation Block the Hippo pathway
AZIN2-sv	CM proliferation ↓	CM proliferation ↓ CM cross-sectional area ↔ Post-MI cardiac function ↑ Post-MI scar area ↓ Post-MI fibrosis ↓	Sponge and protein stabilization Binds and stabilizes PTEN Sponges miR-214

*CM* Cardiomyocyte, *MI* myocardial infarction, *hiPSC* human-induced pluripotent stem cells

Similarly to miRNAs, lncRNAs can regulate cardiomyocyte proliferation either by promoting (positive regulators) or halting (negative regulators) cell cycle progression. Positive regulating lncRNAs are expressed in abundance during heart development to allow for effective cytokinesis and heart growth; however, their expression is undetectable in adulthood. *Endogenous cardiac regeneration-associated regulator (ECRAR)* was shown to be upregulated in human fetal hearts and downregulated during adulthood [24]. ECRAR exhibits a pro-proliferative effect on adult rat cardiomyocytes both in vitro and in vivo. ECRAR overexpression in both P7 and adult post-MI rats resulted in elevated cardiomyocyte proliferation markers and higher capillary density both in the infarct and peri-infarct areas. Additionally, reduced fibrosis and enhanced cardiac function were also observed. In contrast, the knockdown of ECRAR in P1 post-MI rats revealed decreased proliferation and increased fibrosis markers [24]. ECRAR transcription is regulated by E2F transcription factor 1 (E2F1). Acting as a signal lncRNA, ECRAR binds directly to extracellular signal-regulated kinases 1 and 2 (ERK1/2) and accelerates their phosphorylation and translocation to the nucleus where they boost the transcription of several genes involved in cell proliferation. These genes act as activators of cyclin D1 and cyclin E, and increase the expression of E2F1, creating a positive feedback loop [24]. Another positive regulator is NR_045363, which is a mouse homolog of the human LOC101927497. NR_045363 is known to be conserved among various species, including other mammals and birds, suggesting the functional importance of this lncRNA through evolution [142]. This lncRNA is highly expressed in the hearts of embryonic mice and diminishes during adulthood. In vitro, the downregulation of NR_045363 in primary embryonic mouse cardiomyocytes inactivates cell proliferation, and its re-expression in postnatal cardiomyocytes is sufficient to reactivate cardiomyocyte cell cycle progression [142]. Moreover, in in vivo mouse MI models, it was demonstrated that overexpression of NR_045363 resulted in a smaller scar, improved heart function, and enhanced cardiomyocyte proliferation [142]. Mechanistic analyses reveal that NR_045363 acts through direct interaction with miR-216a via a sponge effect. miR-216a is presumed to block the activity of JAK2 [59], a member of the receptor-associated Janus kinase family, which phosphorylates and activates the transcription factor *signal transducer and activator of transcription 3 (STAT3)* [70]. Through the miR-216a/JAK2-STAT3 pathway, lncRNA NR_045363 seems to act as a potent regulator of cardiac cell proliferation [142].

Antisense (AS) lncRNA is a class of lncRNA that is transcribed from the opposite strand of its sense protein-coding gene [26]. An example is *silent information regulator factor 2 related enzyme 1 (Sirt1)* AS which has been reported to induce cell proliferation in various cell types, including myoblasts and endothelial progenitor cells [95, 141]. Recently, a similar effect of Sirt1 AS lncRNA on cardiomyocytes was also reported [81]. Sirt1 AS lncRNA expression was found to be high in mouse embryonic hearts and to decrease after birth. When overexpressed in vitro, Sirt1 AS lncRNA induced mitosis, upregulation of dedifferentiation markers, and a reduction in cell size. When silenced, the opposite results were obtained [81]. Depletion of Sirt1 AS lncRNA in vivo in neonatal mice significantly decreased cardiomyocyte proliferation, while its overexpression in the adult mice induced cell cycle re-activation. When myocardial infarction was induced, Sirt1 AS lncRNA was able to improve survival and cardiac function, decreased scar size, and enhanced proliferation markers [81]. Interestingly, Sirt1 AS lncRNA was found to act by direct binding to Sirt1 mRNA, thus enhancing its stability and increasing the levels of Sirt1 at both the mRNA and protein levels. Moreover, co-transfection with Sirt1 AS lncRNA and small interfering-Sirt1 (which targets Sirt1 mRNA) revealed decreased extent of cardiomyocyte proliferation compared to Sirt1 AS lncRNA alone, suggesting that Sirt1 is involved in Sirt1 AS lncRNA-induced cardiomyocyte proliferation [81].

In contrast to ECRAR, NR_045363, and Sirt1 AS lncRNA, negative regulating lncRNAs are minimally expressed during embryogenesis and development and upregulated in adulthood. *Cardiac regeneration-related lncRNA (CAREL)* is a 748-nucleotide lncRNA that was found to be progressively upregulated in postnatal mice [15]. In the early neonatal stage, cardiomyocyte-specific CAREL transgenic mice and CAREL-adenovirus-transfected mice in which apical resection surgery was performed, exhibited a loss of cardiac regenerative capability, characterized by decreased proliferative potential and hypertrophied cardiomyocytes, increased fibrosis, and altered cardiac function [15]. On the contrary, when CAREL was silenced in adult mice, myocardial infarction resulted in smaller infarct size and improved contractile function [15]. CAREL effects were also investigated in human IPS cell-derived cardiomyocytes. Overexpression of the human conserved sequence of CAREL reduced cell proliferation markers, whereas its knockdown had the opposite effect [15]. Mechanistically, CAREL enhances cardiac regeneration and repair by acting as ceRNA and sponging miRNAs, thus influencing the expression of their target genes. CAREL has a sponge effect on miR-296 which has the ability to induce mitosis and boost cell proliferation by targeting Trp53inp1 and Itm2a whose specific role and function, however, is still unclear [15]. High-throughput sequencing of fetal and adult human cardiac tissue reported differential expression of another lncRNA, NONHSAG007671, proposing it as a negative regulator of cardiomyocyte regeneration [22]. Furthermore, species conservation analysis showed that the exons of NONHSAG007671 are highly conserved across humans, chimps, gorilla, mice, and rats. Designated as *cardiomyocyte regeneration-related lncRNA (CRRL)*, this lncRNA was shown to suppress cardiomyocyte proliferation [22]. In vitro CRRL knockdown studies showed that it facilitated cell proliferation in P1 and P7 rat cardiomyocytes, without changing the cell size [22]. CRRL knockdown in vivo also induced a significant increase in the rate of cardiomyocyte proliferation and resulted in a marked increase in heart weight of both P1 and P7 neonatal rats. In post-MI neonatal and adult rats, blocking CRRL promoted cardiomyocyte proliferation in the peri-infarct area, improved cardiac function, and attenuated post-infarction cardiac remodeling, resulting in a smaller scar area and reduced fibrosis [22]. Conversely, overexpression of CRRL in P1 rats inhibited the post-MI regenerative response. CRRL inhibits cardiomyocyte proliferation by acting as a sponge and absorbing miR-199a-3p. One of the target genes of miR-199a-3p is HOPX [22], which is a critical factor that participates with the enzyme histone deacetylase 2 (Hdac2) in mediating the deacetylation of Gata4 to regulate cardiomyocyte proliferation [135]. These results suggest CRRL as a new potential therapeutic target for heart failure.

Ponnusamy et al. [110] observed that the expression levels of lncRNA AK080084, designated as *Cardiomyocyte Proliferation Regulator (CPR)*, were also low in the embryonic mouse cardiomyocytes, and dramatically increased in postnatal and adult hearts, suggesting that it may play a role in cell cycle arrest in the adult mammalian heart. Cell proliferation assays have shown that silencing of lncRNA CPR in neonatal and adult mouse cardiomyocytes resulted in increased proliferation levels in vivo. On the contrary, CPR overexpression inhibited cardiomyocyte proliferation in neonatal mice and decreased their regenerative capacity after myocardial injury, leading to a larger fibrotic scar. Functionally, the contractile functions of CPR-overexpressed neonatal hearts were significantly depressed after myocardial infarction, while CPR deletion in adult mice resulted in improved left ventricular wall thickness, ejection fraction, and fractional shortening values, with a smaller scar size [110]. Transcriptomic analysis of CPR knockout hearts revealed that 1780 genes were differentially expressed. Among the most upregulated genes was *minichromosome maintenance 3 (MCM3)*, an important factor in initiating eukaryotic genome replication [2]. The authors demonstrated that CPR acts as a guide lncRNA by direct interaction with *DNA methyltransferase 3A (DNMT3A)* to induce MCM3 promoter methylation, thus blocking the cell cycle progression [110]. Another negative regulating lncRNA identified by Cai et al. is lncRNA dachshund homolog 1 (lncDACH1). This 2085-nucleotide, highly conserved lncRNA was previously shown to be involved in the development of heart failure [17], and more recently a prominent role in the regulation of cardiomyocyte proliferation and cardiac regeneration was also reported [16]. When overexpressed in transgenic mice, lncDACH1 reduced proliferation markers expression and increased cardiomyocyte cross-sectional area, suggesting a blockage of mitosis and proliferation mechanisms [16]. Moreover, when apical resection was performed to neonatal transgenic mice, lncDACH1 treatment decreased their heart regenerative capacity. On the contrary, silencing of lncDACH1 enhanced the proliferative potential of cardiomyocytes after ischemic injury in adult mice, and resulted in smaller infarct size, improved cardiac function, and increased proliferation markers [16]. To study the effects of lncDACH1 on human iPSC-derived cardiomyocytes, a conserved sequence of the lncRNA was tested with gain and loss-of-function approaches. Overexpression of lncDACH1 resulted in reduced cardiomyocyte proliferation, while its inhibition promoted cell cycle re-entry [16]. The molecular mechanism underlying the effects of lncDACH1 on cardiac repair was reported to involve a decoy activity by direct interaction with *protein phosphatase 1 alpha (PP1A)* and a reduction in its activity. PP1A enzyme dephosphorylates the transcriptional cofactor YAP1, thereby activating the Hippo transduction pathway which is, as already stated, an essential regulator of cardiomyocyte proliferation [138]. These findings highlight the importance of lncDACH1 in the regulation of the Hippo/YAP pathway, suggesting that, similarly to miRNAs, several lncRNAs can also modulate it.

Finally, AZIN2-sv is a splice variant of the AZIN2 gene that was found to be highly expressed in adult human hearts compared to the fetal stage thus suggesting a negative regulatory role [83]. In vitro, AZIN2-sv was shown to suppress the proliferation genes in cardiomyocytes, while silencing AZIN2-sv resulted in their increase. Overexpression of AZIN2-sv in neonatal rats at P1 and P7 revealed decreased levels of cardiomyocyte proliferation, whereas its knockdown markedly increased these markers and elevated their hearts’ size and weight while the cardiomyocyte cross-sectional area remained unchanged. In post-MI adult rats, reduction in AZIN2-sv resulted in better cardiac function, smaller scars, and less tissue fibrosis [84]. Together, these findings propose a pivotal role of AZIN2-sv in blocking the cell cycle and stop cardiomyocyte proliferation. As to its mechanism of action, AZIN2-sv directly binds PTEN and improves its stability. Additionally, it also sponges miR-214 which has inhibitory effects on PTEN. The overall effect is a significant increase in PTEN levels which results in lower levels of phosphorylated Akt and cyclin D and, consequently, block of the cell cycle [84].

In summary, these studies support the potential of lncRNA-based strategies to boost cardiomyocyte proliferation and cardiac regeneration. The various and intricate regulatory mechanisms are still under intense investigation; however, it is unquestionable that our knowledge of lncRNAs not only adds a new dimension to the molecular architecture of human disease, but also opens up a whole new range of opportunities for future treatments.

## Circular RNAs as targets for cardiomyocyte proliferation

Circular RNA is a special subclass of ncRNA characterized by linking the 3′ and 5′ ends to form a covalently closed loop structure. The circular structure maintains the stability of these ncRNAs, reduces the exonuclease susceptibility, and ultimately results in a longer half-life compared to linear RNA molecules [92]. Recently, circRNAs were shown to be extensively expressed in eukaryotes [145] and to exhibit some degree of evolutionary conservation among mammals [68]. They are dynamically expressed in both tissue development and pathophysiological conditions and they follow tissue- and age-dependent patterns [119, 148]. circRNAs expression is a finely tuned and controlled process involved in various biological roles and for this reason, recent years have witnessed a growing interest in the scientific community.

Different models to describe the modes of action of circRNAs have been proposed. Some circRNAs were found to act as ceRNAs and bind miRNAs [165], whereas others sequester RNA-binding proteins (RBPs) and compete with their mRNA targets [3]. Other proposed modes of action involve competing with the splicing of mRNA [3], interacting directly with regulatory proteins [30], or small nuclear RNA (snRNA) [85] to regulate gene expression, and even being translated to proteins [103].

With the advancement of RNA sequencing technologies, many circRNAs have been confirmed to show abnormal expression in various pathological conditions including cancer [156], neurological [121], and metabolic disorders [146]. circRNAs are also involved in several biological pathological conditions of the cardiovascular system such as cardiomyocyte hypertrophy [143], senescence [29], and apoptosis [157]. Although circRNA investigation in the field of cardiac regeneration and repair has only just started, some encouraging data have already been collected. Circ-Amotl1 is a circular RNA that can boost cardiomyocyte survival following in vitro exposure to H_2_O_2_. It was also shown to attenuate doxorubicin-induced cardiomyopathy in mice by binding phosphoinositide-dependent kinase 1 (PDK1) and AKT1, resulting in AKT1 phosphorylation and nuclear translocation [157]. Another example is circFndc3b which harbor cardioprotective effects by reducing cardiomyocyte apoptosis following in vitro stress conditions such as exposure to H_2_O_2_ serum deprivation and hypoxic stress. In vivo data also demonstrated a reduction in cardiomyocyte apoptosis and improved neovascularization and left ventricular functions post-MI in circFndc3b treated animals [46].

Very limited scientific evidence is currently available to robustly support the contribution of circRNAs in the regulation of cardiomyocyte cell cycle. So far, only one circRNA was demonstrated to regulate cardiac myocyte proliferation. CircRNA Nfix is a super enhancer-associated circRNA that is highly conserved in humans, rats, and mice, and was found to be enriched in adult cardiomyocytes. When overexpressed in neonatal mice, circNfix reduced both proliferation and angiogenesis. Overexpression in MI neonatal mice decreased cardiomyocyte proliferation rate, ultimately resulting in impaired cardiac function and larger infarct size. On the contrary, the loss of circNfix stimulated cardiomyocyte proliferation in vitro and resulted in improved cell survival after exposure to H_2_O_2_ treatment. Moreover, circNfix silencing in adult mice induced enhanced cardiomyocytes number and proliferation capacity, together with increased arteriolar density and decreased apoptosis, ultimately resulting in improved cardiac function and reduced infarct area and cardiac fibrosis [63]. CircNfix was found to be regulated by Meis1, a key transcription factor controlling cardiomyocyte cell cycle arrest, which binds and activates the super-enhancer region of circNfix. CircNfix was suggested to act by binding to the transcription factor Ybx1 while recruiting the E3 ubiquitin ligase Nedd4l, thereby mediating the arrest of Ybx1 in the cytoplasm and its degradation through ubiquitination–proteasome pathways. Nuclear Ybx1 is known to bind to the promoters of CCNA2 (cyclin A2) and CCNB1 (cyclin B1) and activate their transcription, facilitating cell cycle entry [72]. In parallel, circNfix’s proangiogenic activity is mediated by sponging of miR-214, thus inhibiting Gsk3β expression. CircNfix/miR-214/Gsk3β pathway regulates VEGF release from cardiomyocytes and regulates the formation of blood vessels.

As demonstrated by the limited literature, circRNAs research particularly in the regenerative medicine field is still in its infancy, and because of its potential gaining increasing interest from the scientific community. The deeper understanding of circRNAs mechanisms and development of new technologies will further contribute to this field providing new insights and therapeutic possibilities for the treatment of heart failure.

## Current limitations and future insights

The field of cardiac regeneration has made enormous progress and various regulatory networks that control cardiomyocyte proliferation have been unravelled. Several methodological and technological limitations still remain. For a long time, the field has been enfeebled by the improper use of techniques that claim to indicate cardiomyocyte proliferation. Ki-67, phosphohistone H3 (pH3), Aurora B, and nucleotide analogs are examples of immunohistochemical markers that are frequently used to demonstrate cell cycle activity [15, 17, 24, 34, 81, 84, 132]. These methods, however, have crucial limitations which, if not considered, can result in misleading data interpretation. Nucleotide analogs are incorporated into the newly synthesized DNA and can be easily detected by immunofluorescent staining [49]. Cardiomyocytes, however, undergo various processes such as endoreduplication, endomitosis, and multinucleation, which imply DNA synthesis but do not result in complete cell division. pH3 is also a popular marker which labels phosphorylated histone H3 that is believed to be crucial for chromosome condensation and cell cycle progression during mitosis [58]. Histone H3 phosphorylation occurs from late G2 phase to early telophase [57]. Similarly to nucleotide analogs, this assay is not sufficient to indicate cell proliferation, as cells undergoing endomitosis or multinucleation will also be labeled [31, 91]. Ki67 is a protein required for the formation of the perichromosomal layer and plays a key role in preventing the aggregation of mitotic chromosomes [128]. It is clearly required for cell proliferation, but it is also re-expressed in cardiomyocytes undergoing hypertrophy, endoreduplication, and endomitosis [91]. Taken together, these considerations suggest that a composite approach which includes the use of various markers, that distinguish different parts of the cell cycle, in combination with other approaches such as cell proliferation assays, cell count, and clonal analysis, can provide a more reliable readout.

Another challenge that still needs to be addressed is the development of strategies that can modulate cardiomyocyte proliferation rate as a mean of increase but also limit this process. Uncontrolled proliferation can indeed result in pathological conditions such as uncontrolled hypertrophy and enlarged myocardium. Several studies on both rodents and pigs have reported adverse effects in response to prolonged treatment with various miRNAs; these included cardiomegaly, impaired cardiac function, arrhythmic events and sudden death [41, 132]. An additional aspect that remains to be verified is the actual impact that enhanced cardiomyocyte proliferation can play in the context of the whole heart. Several studies have reported significant improvements in cardiac function and structure which were attributed to this process. However, in various cases a minor increase in cardiomyocyte cell cycle activity was demonstrated, which was not sufficient to justify the overall beneficial effects [16, 122, 142]. Other factors such as enhanced angiogenesis, hypertrophy, reduced apoptosis, or altered metabolic activity may also contribute and, thus, should be considered.

Furthermore, humans have both genetic and physiological features that are different from other species, and which are only partially recapitulated by research models. These aspects are certainly confounding factors and they contribute to complicating the translation of findings into human applications. A clear example is lncRNAs, as they are poorly conserved among species; therefore, humanized models or organoid cultures are often required for translatability of finding to human biology [81]. Moreover, accumulating evidence seems to indicate the existence of a specific subpopulation of mononuclear diploid cardiomyocytes in the murine hearts which contribute widely to new cardiomyocyte formation [75, 106]. Thus, it appears important to determine whether this subpopulation of cardiomyocytes exists in humans and other mammals and how it could be targeted and used to boost regenerative processes. Finally, it should be considered that the myocardium consists of several cell types that communicate and interact in a variety of ways [133]. Thus, it seems essential to elucidate the associations between these multicellular interactions and regenerative processes. A better understanding of this area could be exploited to control and boost cardiomyocyte proliferation.

Although several challenges remain, the field of cardiomyocyte proliferation has made enormous steps forward and ncRNAs hold tremendous promise for regenerative medicine. A recent translational study, in a large animal model, indicated improved contractility, increased muscle mass, and reduced post-MI scar size in response to miRNA treatment [41]. To date, no clinical trials have been held to investigate the proliferative potential of ncRNAs in the heart; however, preclinical development of an inhibitor for the pro-hypertrophic miR-132 has shown favorable pharmacokinetics, safety, and tolerability of the drug, now leading to a first phase 1b clinical trial in heart failure patients [62]. These findings suggest the enormous clinical therapeutic potential for miRNA mimics and inhibitors and more preclinical and clinical studies can be expected in the near future.

## Conclusions

The field of cardiac regeneration has been exploring numerous avenues to achieve effective cardiac repair, and being able to restore (and control) the proliferative capacity of adult cardiomyocytes is possibly the biggest challenge in regenerative medicine. The information collected and summarized in this review indicates that cardiomyocytes have an intrinsic capacity to proliferate which, in the steady-state, is suppressed to various extents by either extra- or intracellular factors. The nature and importance of such stimuli and how they impact the cardiomyocyte cell cycle are still under intensive investigation. The understanding of the regenerative capacity of the heart along evolution has been essential to improve our knowledge of the cellular mechanisms which are often shared between lower vertebrates and neonatal mammals. This holds promise as the cardiomyogenesis regulation mechanisms identified in these animal models seem to act in a similar manner in humans. Both short and long ncRNAs are primary orchestrators of crucial gene regulatory networks controlling various cellular processes. miRNAs experimentation is leading the research and it is ahead in comparison to other ncRNAs. lncRNAs remain an immense, poorly explored, and inadequately understood source for new regenerative targets. Recent advancements in sequencing have allowed large-scale target identification and the new gene therapy technologies provide new tools for their precise in vivo modulation. These are crucial requirements to elucidate the regulatory networks implicated in cell cycle activation and arrest. Micro, circular, and lncRNAs were shown to have extremely versatile modes of action and to be expressed abundantly in the heart tissue, suggesting that they are an appealing target for future cardiac regenerative research. The mechanisms that underlie the activity of some of the described long non-coding and circular RNAs seem to largely overlap with pathways that were shown being essential in microRNA regulation, suggesting that a more refined and possibly combined approach might lead to more effective strategies. Understanding and taking advantage of the mechanisms will eventually help to remove and control the breaks on myocardial regeneration.
